# A survey of the European Society of Clinical Pharmacy members’ research involvement, and associated enablers and barriers

**DOI:** 10.1007/s11096-020-01054-9

**Published:** 2020-05-19

**Authors:** Derek Stewart, Vibhu Paudyal, Cathal Cadogan, Ankie Hazen, Betul Okuyan, Monika Lutters, Martin Henman, Daniella Fialová

**Affiliations:** 1 College of Pharmacy, QU Health, Qatar University, Doha, Qatar; 2 School of Pharmacy, University of Birmingham, Birmingham, UK; 3 School of Pharmacy and Biomolecular Sciences, Royal College of Surgeons in Ireland, Dublin, Ireland; 4 Centre for Pharmacy Postgraduate Education, The University of Manchester, Manchester, UK; 5 Marmara University, Istanbul, Turkey; 6 Kantonsspital Baden, Baden, Switzerland; 7 The School of Pharmacy and Pharmaceutical Sciences, Trinity College Dublin, Dublin, Ireland; 8 Charles University in Prague, Prague, Czech Republic; 9 Department of Geriatrics and Gerontology, 1st Faculty of Medicine, Prague, Czech Republic

**Keywords:** Barriers, Clinical pharmacy, Enablers, European Society of Clinical Pharmacy, Research

## Abstract

*Background* Building research capacity of European Society of Clinical Pharmacy (ESCP) members aligns to the organisation’s aim of advancing research. *Objective* To determine members’ aspirations and needs in research training and practice, and to explore ways in which ESCP could provide support. *Setting* ESCP’s international membership. *Method* Cross-sectional survey of members in 2018, followed by focus groups with samples of respondents attending an ESCP symposium. Survey items were: research activities; interests, experience and confidence; and Likert statements on research conduct. Principal component analysis (PCA) clustering of Likert statements from a previous study was used, with scores for each component calculated. Focus groups discussed barriers to research and how ESCP could provide support. Data analysis involved collating and comparing all themes. *Main outcome measures* Research interest, experience and confidence; attitudinal items; barriers to research; ESCP support. *Results* The response rate was 16.7% (83/499), with 89.2% (n = 74) involved in research and 79.5% (n = 66) publishing research in the preceding 2 years. While overwhelmingly positive, responses were more positive for research interest than experience or confidence. PCA component scores (support/opportunities, motivation/outcomes, and roles/characteristics) were positive. Thirteen members participated in focus groups, identifying barriers of: insufficient collaboration; lack of knowledge, skills, training; unsupportive environment; insufficient time; and limited resources. ESCP could support through mentorship, collaboration, education and funding. *Conclusion* Study participants were highly active, interested, experienced, confident and positive regarding research. There is an opportunity for ESCP to harness these activities and provide support in the form of mentoring, education and training, and facilitating collaboration.

## Impacts on practice

- There is an opportunity for ESCP to harness the research activities, interest, experience, confidence and generally positive views to create greater impact for patients, professionals, organisations and society across Europe and beyond.
- ESCP as an organisation can provide support in the form of mentoring, education and training, focusing most on those form non-academic settings.

## Introduction

Pharmacy practice is continually evolving, with pharmacists providing enhanced services involving direct patient care, such as general practice-based activities, non-medical prescribing, and specialist roles in ambulatory, secondary and tertiary care [1–4]. Consequently, the application of evidence-based practice (EBP), the integration of best available evidence with practitioners’ clinical expertise and patients’ values [5], is highly relevant. Awareness of, and participation in, research could facilitate adoption of EBP within current and emerging roles.

Professional organisations and regulatory bodies are placing increasing emphasis on participation in research. For example, the International Pharmaceutical Federation (FIP) emphasises the need for pharmacists to take on significant responsibilities in research in order to contribute to improvements in global health by advancing drug discovery and development, clinical practice and education [6].

However, limited published research exists on pharmacists’ current involvement in research and their future needs. Current available literature is mostly based in either the UK or non-European countries [7, 8]. In addition, there is a lack of consistent application of how research involvement or engagement has been defined and adopted. The existing published literature, albeit limited, suggests that barriers to participation and involvement are often behavioural in nature. For example, barriers include a lack of knowledge, skills and self-motivation. There is therefore a need to incorporate behaviour change theories or frameworks in undertaking future research in the area. Incorporation of behaviour change theory permits the identification of possible theoretical mechanisms of behaviour change leading to the development of targeted intervention(s) [9]. The Theoretical Domains Framework (TDF) includes constructs from 33 behaviour change theories, described in 14 domains (i.e. the determinants of behaviour) of: knowledge; skills; social/professional role and identity; beliefs about capabilities; optimism; beliefs about consequences; reinforcement; intentions; goals; memory, attention and decision processes; environmental context and resources; social influences; emotions; and behavioural regulation.

The use of the TDF has previously enabled assessment of associated barriers of pharmacists’ involvement in research, in diverse practice settings in Scotland [8]. Key barriers mapped to TDF domains of ‘knowledge’ (e.g. organization research priorities, training and funding opportunities), and ‘environmental context and resources’ (e.g. research active environment, time, support). Study limitations included the low response rate and absence of a qualitative phase to allow in-depth exploration of survey findings.

The European Society of Clinical Pharmacy (ESCP) is a professional network of clinical pharmacists in Europe. Established in 1979, ESCP aims to ‘promote, support, implement and advance education, practice and research in clinical pharmacy in order to optimise outcomes for patients and society’ [10]. Building research capacity, defined as ‘enhancing the abilities of individuals, organisations and systems to undertake and disseminate high quality research effectively and efficiently’ [11], aligns to this aim. There is an opportunity to engage with this network to identify the needs and future requirement of pharmacists in order to harness pharmacy practice research capacity and activities across Europe.

### Aim of the study

Aims of the study were to determine ESCP members’ aspirations and needs in relation to research training and practice and to explore ways in which ESCP could support members in meeting these needs.

### Ethics approval

The ESCP General Committee approved the study; as participants were members of an international organisation, there was no need for ethical review.

## Method

### Design

This was an explanatory, sequential mixed methods study involving a cross-sectional survey followed by a qualitative phase.

### Cross-sectional survey

#### Inclusion and exclusion criteria

All ESCP members were invited to participate, excluding members of the ESCP Research Committee (n = 8), giving a study population of 499.

#### Questionnaire development

The questionnaire was based on that used in Scotland [8], adapted for the ESCP context. Items were based on the 14 domains of the TDF and the Transtheoretical Model of Behaviour Change [12]. Stages of change are ‘pre-contemplation’ (not ready), ‘contemplation’ (getting ready), ‘preparation’ (ready), ‘action’ and ‘maintenance’. In the demographics section, respondents classified themselves as innovators, early adopters, early majority, late majority and laggards based on receptivity to change, using the wording described by Rogers [13].

The questionnaire was tested for face and content validity by the members of the ESCP Research Committee. Given the previous use of a similar questionnaire [8], no pilot stage was conducted. The questionnaire was developed in Bristol Online Survey© and tested for compatibility with platforms (PC, tablet, smartphone), browsers and internet filters. Question types were a combination of closed and open questions to allow respondents to provide comments. Evidence-based strategies were employed to maximise the response rate [14], including: an information leaflet outlining the study aim and potential benefits; assuring anonymity; an attractive questionnaire; and two follow-up email reminders.

#### Data collection

During September 2018, ESCP members were sent an email from the ESCP International Office with a direct link to the information leaflet and questionnaire. Two email reminders were sent to all ESCP members at approximately 2-monthly intervals.

### Analysis

The survey instrument generated data that were exported to Statistical Package for Social Sciences (SPSS Inc., Cary, NC version 25.0). Descriptive analysis was undertaken for: demographics; research activities; research interest, experience and confidence; views on research conduct; and readiness to participate in research and research training. Internal consistencies of responses on interest, experience and confidence were tested using Cronbach’s alpha, aiming for values ≥ 0.7 [15]. Total scores [median and interquartile range (IQR)] for each scale were obtained by assigning values (1 = no to 5 = very). Differences in total scores of interest, experience and confidence were tested using Friedman’s two-way analysis of variance by ranks. Correlation between overall scores of interest/experience, interest/confidence and experience/confidence were assessed using Spearman’s rho. *P* values ≤ 0.05 were considered to be statistically significant. Given the number of responses, it was not possible to conduct any inferential statistical analysis to investigate any differences between subgroups (e.g. age etc.)

While it was intended to use principal component analysis (PCA) to reduce the large number of TDF-related items to a smaller number of components [16], the number of responses proved insufficient. Consequently, the PCA components identified in the study in Scotland [8], were used to group the items into: support and opportunities to be involved in research; motivation for and outcomes of involvement in research; and individual roles and characteristics around involvement in research. Following determination of internal consistencies for each of the three components, total scores (median and interquartile range, IQR) were obtained by assigning scores of 1 (strongly disagree) to 5 (strongly agree) to each of the Likert statement responses and each of these compared to the scale midpoint.

Summative content analysis was independently performed by two members of the research team on the responses to the open questions, looking for patterns, similarities and differences [17].

### ESCP symposium focus group

#### Recruitment

The focus group discussion took place at the ESCP Symposium in October 2018. Those attending the symposium who had completed the questionnaire were invited to participate. The session was of 1-h duration and moderated by three ESCP Research Committee members.

#### Data generation and analysis

Participants were arranged into groups, with data generated in two separate stages. In Stage 1, each participant was asked to individually record the top three barriers to fulfilling their research aspirations. These were recorded on post-it notes, which were then arranged into thematic groupings on a flip chart by group members. In Stage 2, groups discussed how ESCP could facilitate their research aspirations, being cognisant of the limited resources of ESCP. Each group recorded the outcomes of their discussion on flip charts as in Stage 1. Data analysis was independently undertaken by two researchers, involving collating, comparing and contrasting all themes.

## Results

### Cross-sectional survey

#### Demographics

Eighty-three responses were received, giving a response rate of 16.7%. Personal and practice demographics are shown in Table 1. Respondents were from a range of largely European countries, mainly Switzerland (13.3%, n = 11), Netherlands (10.8%, n = 9), Norway (7.2%, n = 6) and the UK (7.2%, n = 6). The majority had been ESCP members for ≤ 5 years (55.4%, n = 46), were aged < 50 years (71.1%, n = 59) and male (65.1%, n = 54). Just under half were working in academia (42.2%, n = 35), had worked > 15 years (47.0%, n = 39) and the majority were working full-time (81.9%, n = 68). Respondents were highly qualified with more than half possessing a Ph.D. (68.7%, n = 57). In terms of receptivity to change, almost three quarters (72.3%, n = 70) classified themselves as innovators and early adopters.

**Table 1:** Personal and practice demographics of respondents (n = 83)

Demographic	% (n)
Age (years)	
21–30	13.3 (11)
31–40	31.3 (26)
41–50	26.5 (22)
51–60	15.7 (13)
> 60	13.3 (11)
Gender	
Female	34.9 (29)
Male	65.1 (54)
Country of practice	
Switzerland	13.3 (11)
Netherlands	10.8 (9)
Norway	7.2 (6)
United Kingdom	7.2 (6)
Belgium	6.0 (5)
France	6.0 (5)
Germany	6.0 (5)
Italy	4.8 (4)
Malta	3.6 (3)
Portugal	3.6 (3)
Turkey	3.6 (3)
Greece	2.4 (2)
Serbia	2.4 (2)
Slovakia	2.4 (2)
Sweden	2.4 (2)
Albania, Croatia, Cyprus, Denmark, Estonia, ‘EU’, Ireland, Spain	9.6 (1 each)
Asia	8.4 (7)
Africa	1.2 (1)
Years member of ESCP	
≤ 5	55.4 (46)
6–10	12.0 (10)
11–15	13.3 (11)
16–20	6.0 (5)
21–25	7.2 (6)
26–30	3.6 (3)
> 30	2.4 (2)
Main practice setting	
Academia	42.2 (35)
University hospital	22.9 (19)
Non-university hospital	14.5 (12)
Community pharmacy	7.2 (6)
Industry	1.2 (1)
Primary care medical practice	1.2 (1)
Other	10.8 (9)
Years of working as pharmacist (excluding career breaks)	
0	4.8 (4)
1–5	18.1 (15)
6–10	12.0 (10)
11–15	18.1 (15)
16–20	10.8 (9)
21–25	18.1 (15)
26–30	4.8 (4)
> 30	13.3 (11)
Working schedule	
Full-time	81.9 (68)
Part-time	9.6 (8)
Other	8.4 (11)
Postgraduate qualifications	
Ph.D.	68.7 (57)
Masters	38.6 (32)
Diploma	21.7 (18)
Certificate	22.9 (19)
Receptivity to change	
Innovative with new ways of working (innovator)	55.4 (46)
Serve as role model for others (early adopter)	16.9 (14)
Think for some time before adopting new ways of working (early majority)	26.5 (22)
Cautious, tend to change once most peers have done so (late majority)	0
Resist new ways of working (laggard)	1.2 (1)

#### Research involvement

Over the preceding 2 years, 89.2% (n = 74) had been involved in research, 51.8% (n = 43) as principal investigator and 54.2% (n = 45) as co-investigator. The majority (79.5%, n = 66) had published research, 45.8% (n = 38) as corresponding author and 59.0% (n = 49) as co-author. Three quarters (74.7%, n = 62) had presented research at an international conference and around two-fifths (38.6%, n = 32) had supervised doctoral students.

#### Research interest, experience and confidence

Responses to items on interest, experience and confidence are reported in Table 2. The Cronbach’s alpha values for the items on interest, experience and confidence were 0.91, 0.96 and 0.95 respectively, indicating internal reliability. The median summary score (range possible 16–80, scale mid-point 48; high scores positive) for research interest was 68 (IQR 59–73), research experience 62 (IQR 53–70) and research confidence 64 (IQR 56–70). Summary scores for interest were significantly higher than confidence which were significantly higher than experience (χ^2^, *P* < 0.001). There were positive correlations between total scores for interest and experience, interest and confidence, and confidence and experience (Spearman’s rho, *P* < 0.001), with those more interested also more experienced and confident. The most negative responses were for items on: interest in conducting a systematic review, 42.1% (n = 35) reported no/little/some interest; experience in conducting a systematic review, 59.1% (n = 49) reported no/little/some experience; confidence in conducting a systematic review, 49.4% (n = 41) reported no/little/some confidence; and confidence in analysing and interpreting qualitative results, 47.0% (n = 39) reported no/little/some confidence.

**Table 2:** Responses to items of interest, experience and confidence in specific aspects of research (n = 83)

Item	Interest % (n)					Experience % (n)					Confidence % (n)				
	No interest	Little interest	Some interest	Moderate interest	Very interested	No experience	Little experience	Some experience	Moderate experience	Very experienced	No confidence	Little confidence	Some confidence	Moderate confidence	Very confident
Research advances within my field and in related areas	0	3.6 (3)	2.4 (2)	13.3 (11)	80.7 (67)	4.8 (4)	4.8 (4)	15.7 (13)	51.8 (43)	22.9 (19)	2.4 (2)	4.8 (4)	16.9 (14)	45.8 (38)	28.9 (24)
Generating research ideas	0	3.6 (3)	15.7 (13)	16.9 (14)	63.9 (53)	6.0 (5)	1.2 (1)	18.1 (15)	42.2 (35)	32.5 (27)	0	10.8 (9)	13.3 (11)	47.0 (39)	28.9 (24)
Developing research questions, aims, hypotheses and objectives	1.2 (1)	7.2 (6)	13.3 (11)	19.3 (16)	59.0 (49)	4.8 (4)	2.4 (2)	18.1 (15)	45.8 (38)	28.9 (24)	3.6 (3)	4.8 (4)	19.3 (16)	44.6 (37)	27.7 (23)
Finding relevant literature	1.2 (1)	3.6 (3)	16.9 (14)	30.1 (25)	48.2 (40)	1.2 (1)	4.8 (4)	12.0 (10)	42.2 (35)	39.8 (33)	0	7.2 (6)	7.2 (6)	51.8 (43)	32.5 (27)
Reviewing literature	0	3.6 (3)	25.3 (21)	41.0 (34)	30.1 (25)	0	8.4 (7)	21.7 (18)	38.6 (32)	31.1 (26)	1.2 (1)	7.2 (6)	9.6 (8)	44.6 (37)	36.1 (30)
Writing a research proposal	1.2 (1)	12.0 (10)	15.7 (13)	31.3 (26)	39.8 (33)	8.4 (7)	6.0 (5)	22.9 (19)	43.4 (36)	19.3 (16)	6.0 (5)	8.4 (7)	16.9 (14)	55.4 (46)	13.3 (11)
Conducting a systematic review	4.8 (4)	12.0 (10)	25.3 (21)	34.9 (29)	22.9 (19)	16.9 (14)	22.9 (19)	19.3 (16)	28.9 (24)	12.0 (10)	7.2 (6)	21.7 (18)	20.5 (17)	33.7 (28)	15.7 (13)
Using quantitative research methods (e.g. RCTs, cohort studies, surveys, questionnaires)	1.2 (1)	2.4 (2)	16.9 (14)	41.0 (34)	38.6 (32)	4.8 (4)	12.0 (10)	15.7 (13)	48.2 (40)	19.3 (16)	1.2 (1)	15.7 (13)	15.7 (13)	45.8 (38)	20.5 (17)
Using qualitative research methods (e.g. focus groups, interviews)	3.6 (3)	8.4 (7)	15.7 (13)	41.0 (34)	31.3 (26)	13.3 (11)	14.5 (12)	16.9 (14)	37.3 (31)	18.1 (15)	6.0 (5)	14.5 (12)	26.5 (22)	36.1 (30)	16.9 (14)
Analysing and interpreting quantitative results	2.4 (2)	3.6 (3)	18.1 (15)	36.1 (30)	39.8 (33)	6.0 (5)	6.0 (5)	16.9 (14)	53.0 (44)	18.1 (15)	4.8 (4)	10.8 (9)	14.5 (12)	48.2 (40)	21.7 (18)
Analysing and interpreting qualitative results	1.2 (1)	8.4 (7)	22.9 (19)	39.8 (33)	27.7 (23)	12.0 (10)	12.0 (10)	20.5 (17)	42.2 (35)	13.3 (11)	7.2 (6)	15.7 (13)	24.1 (20)	33.7 (28)	18.1 (15)
Giving an oral presentation in my practice setting	1.2 (1)	7.2 (6)	16.9 (14)	24.1 (20)	50.6 (42)	1.2 (1)	3.6 (3)	12.0 (10)	30.1 (25)	53.0 (44)	0	6.0 (5)	10.8 (9)	28.9 (24)	53.0 (44)
Giving an oral presentation at national or international conference	0	6.0 (5)	14.5 (12)	24.1 (20)	55.4 (46)	4.8 (4)	12.0 (10)	12.0 (10)	27.7 (23)	43.4 (36)	2.4 (2)	8.4 (7)	10.8 (9)	34.9 (29)	43.4 (36)
Writing and publishing research in academic journals	2.4 (2)	3.6 (3)	12.0 (10)	25.3 (21)	56.6 (47)	9.6 (8)	7.2 (6)	14.5 (12)	43.4 (36)	25.3 (21)	4.8 (4)	10.8 (9)	12.0 (10)	47.0 (39)	24.1 (20)
Reading and interpreting research	1.2 (1)	2.4 (2)	18.1 (15)	31.3 (26)	47.0 (39)	1.2 (1)	6.0 (5)	13.3 (11)	51.8 (43)	27.7 (23)	1.2 (1)	3.6 (3)	18.1 (15)	43.4 (36)	32.5 (27)
Applying the outcomes of research to your practice	0	3.6 (3)	6.0 (5)	31.3 (26)	59.0 (49)	3.6 (3)	8.4 (7)	21.7 (18)	45.8 (38)	20.5 (17)	1.2 (1)	7.2 (6)	27.7 (23)	31.3 (26)	32.5 (27)
Scale statistics, sum of allocating 1 (no interest, experience, confidence) to 5 (very interested, experienced, confident)	Cronbach’s alpha 0.91					Cronbach’s alpha 0.96					Cronbach’s alpha 0.95				
	Range possible 16–80					Range possible 16–80					Range possible 16–80				
	Mid-point 48					Mid-point 48					Mid-point 48				
	Median 68					Median 62					Median 64				
	IQR 59–73					IQR 53–70					IQR 56–70				

Responses do not all total 100% due to missing data

### Research involvement

Attitudinal statements were grouped into three components as per the previous study [8], labelled: support and opportunities to participate in research (Cronbach’s alpha 0.90); motivation for and outcomes of participation in research (Cronbach’s alpha 0.896); and individual roles and characteristics around participation in research (Cronbach’s alpha 0.82). Responses to items of these three components are given in Table 3.

**Table 3:** Responses to attitudinal items on aspects of research conduct (n = 83)

Statement	Strongly agree % (n)	Agree % (n)	Unsure % (n)	Disagree % (n)	Strongly disagree % (n)
*Component 1: Support and opportunities to be involved in research*					
I work within a research-supportive environment	26.5 (22)	44.6 (37)	15.7 (13)	12.0 (10)	1.2 (1)
I work within a research-active environment	30.1 (25)	38.6 (32)	14.5 (12)	14.5 (12)	2.4 (2)
I am fully aware of the research priorities for my organization	24.1 (20)	48.2 (40)	22.9 (19)	3.6 (3)	1.2 (1)
Being involved in research is/would be supported by my organisation	43.4 (36)	36.1 (30)	16.9 (14)	3.6 (3)	0
Being involved in research is/would be supported by my profession	41.0 (34)	43.4 (36)	14.5 (12)	1.2 (1)	0
Being involved in research is/would be supported by my line manager	36.1 (30)	38.6 (32)	18.1 (15)	4.8 (4)	2.4 (2)
Being involved in research is/would be supported by my peers	38.6 (32)	36.1 (30)	20.5 (17)	4.8 (4)	0
I am fully aware of training opportunities relating to research	19.3 (16)	43.4 (36)	31.3 (26)	4.8 (4)	1.2 (1)
I am fully aware of funding opportunities relating to research	8.4 (7)	36.1 (30)	42.2 (35)	12.0 (10)	1.2 (1)
I am fully aware of opportunities to be involved in research	24.1 (20)	54.2 (45)	19.3 (16)	0	2.4 (2)
I have clear goals for being involved in research	44.6 (37)	39.8 (33)	9.6 (8)	4.8 (4)	1.2 (1)
Other pharmacists I know influence me to be involved in research	22.9 (19)	44.6 (37)	15.7 (13)	15.7 (13)	1.2 (1)
Other health professionals I know influence me to be involved in research	22.9 (19)	48.2 (40)	16.9 (14)	10.8 (9)	1.2 (1)
I have sufficient time to participate in research	13.3 (11)	31.3 (26)	20.5 (17)	22.9 (19)	12.0 (10)
I have access to all of the resources (e.g. statistical advice, software) I need to be involved in research	20.5 (17)	30.1 (25)	25.3 (21)	21.7 (18)	2.4 (2)
There are opportunities for me to attend research talks and seminars	24.1 (20)	56.6 (47)	12.0 (10)	7.2 (6)	0
There are opportunities for me to attend national research conferences	32.5 (27)	47.0 (39)	15.7 (13)	4.8 (4)	0
There are opportunities for me to attend international research conferences	27.7 (23)	50.6 (42)	16.9 (14)	4.8 (4)	0
Component statistics, sum of allocating 1 (strongly disagree) to 5 (strongly agree)					
Cronbach’s alpha 0.90					
Range possible 18–90, with 90 representing best positive attitudinal score					
Mid-point 54					
Median 70					
IQR 61–77					
*Component 2: Motivation for and outcomes of involvement in research*					
Being involved in research is/would be of benefit to my profession	77.1 (64)	21.7 (18)	1.2 (1)	0	0
Being involved in research is/would be of benefit to my career	57.8 (48)	31.3 (26)	6.0 (5)	3.6 (3)	1.2 (1)
Being involved in research is/would be of benefit to patients	53.0 (44)	36.1 (30)	9.6 (8)	0	0
Being involved in research is/would be of benefit to my organisation	59.0 (49)	32.5 (27)	7.2 (6)	0	0
Being involved in research is/would be of benefit to my professional practice	63.9 (53)	30.1 (25)	4.8 (4)	0	0
I get/would get professional satisfaction from being involved in research	62.7 (52)	32.5 (27)	3.6 (3)	1.2 (1)	0
I am motivated to be involved in research	60.2 (50)	31.3 (26)	6.0 (5)	2.4 (2)	0
Component statistics, sum of allocating 1 (strongly disagree) to 5 (strongly agree)					
Cronbach’s alpha 0.86					
Range possible, 7–35 with 35 representing best positive attitudinal score					
Mid-point 21					
Median 33					
IQR 29–35					
*Component 3: Individual roles and characteristics around involvement in research*					
*Only academics should be involved in research	2.4 (2)	2.4 (2)	2.4 (2)	43.4 (36)	48.2 (40)
Being involved in research is already part of my professional role	57.8 (48)	33.7 (28)	3.6 (3)	3.6 (3)	0
It is my professional duty to be involved in research	57.8 (48)	24.1 (20)	13.3 (11)	2.4 (2)	2.4 (2)
I have sufficient methodological knowledge to be involved in research	33.7 (28)	49.4 (41)	14.5 (12)	1.2 (1)	1.2 (1)
I am sufficiently skilled to be involved in research	43.4 (36)	44.6 (37)	10.8 (9)	1.2 (1)	0
I am able to determine my own research-related training needs	36.1 (30)	50.6 (42)	9.6 (8)	3.6 (3)	0
I am confident in my ability to participate in research	47.0 (39)	44.6 (37)	8.4 (7)	0	0
I am competent to be involved in research	47.0 (39)	44.6 (37)	8.4 (7)	0	0
*I feel anxious about being involved in research	6.0 (5)	14.5 (12)	12.0 (10)	41.0 (34)	25.3 (21)
I am enthusiastic about being involved in research	57.8 (48)	36.1 (30)	3.6 (3)	2.4 (2)	0
Being involved in research makes/would make me feel happy	48.2 (40)	39.8 (33)	12.0 (10)	0	0
I support others to be involved in research	57.8 (48)	36.1 (30)	6.0 (5)	0	0
*Negative statement, reverse scored					
Component statistics, sum of allocating 1 (strongly disagree) to 5 (strongly agree)					
Cronbach’s alpha 0.82					
Range possible 12–60, with 60 representing best positive attitudinal score					
Mid-point 36					
Median 52					
IQR 47–56					

Responses do not all total 100% due to missing data

#### Component 1: Support and opportunities to be involved in research

Respondents generally held positive views, with a median overall score of 70 (IQR 61–77), range possible 18–90 (midpoint 54), with 85 representing the highest possible positive score. The statements with the highest levels of disagreement were for the statements, ‘I have sufficient time to participate in research’ (disagree/strongly disagree 34.9%, n = 29) and ‘I have access to all of the resources (e.g. statistical advice, software) I need to be involved in research’ (disagree/strongly disagree 24.1%, n = 20).

#### Component 2: Motivation for and outcomes of involvement in research

Responses were very positive, with a median overall score of 33 (IQR 29–35), range possible 7–35 (midpoint 21), with 35 representing the highest possible positive score. The statements with the highest levels of agreement were for the statements, ‘Being involved in research is/would be of benefit to my profession’ (agree/strongly agree 98.8%, n = 82) and ‘I get/would get professional satisfaction from being involved in research’ (agree/strongly agree 95.1%, n = 79).

#### Component 3: Individual roles and characteristics around involvement in research

Respondents generally held very positive views, with a median overall score of 52 (IQR 47–56), range possible 12–60 (midpoint 36), with 60 representing the highest possible positive score. The most negative response was for ‘I feel anxious about being involved in research’ (agree/strongly agree 20.5%, n = 17).

### Readiness to be involved in research

In response to questions on readiness to be involved in research: 1.2% (n = 1) had never thought about being involved in research; 6.0% (n = 5) had thought about being involved but had taken no action; 3.6% (n = 3) had thought about being involved and discussed with others; 7.2% (n = 6) had been involved in research in the past but had no plans to be involved in the future; 10.8% (n = 9) had been involved in research in the past and had plans to be involved in the future; and 71.1% (n = 59) were currently involved in research.

In terms of research training: 6.0% (n = 5) had never thought about research training; 14.5% (n = 12) had thought about research training but had taken no action; 16.9% (n = 14) had thought about research training and discussed with others; 6.0% (n = 5) had a plan for their research training; 6.0% (n = 5) had enrolled for research training; and 50.6% (n = 42) had undertaken research training.

Relating to the specific types of training: 9.6% (n = 8) were not interested in any training; 34.9% (n = 29) were interested in training but not leading to a formal university qualification; 16.9% (n = 14) were interested in university training at postgraduate certificate or postgraduate diploma levels; 4.8% (n = 4) were interested in university training at masters level; and 33.7% (n = 28) were interested in university training at doctoral level.

Content analysis of responses to open questions throughout the questionnaire identified several key themes. While many expressed a desire to be more involved in research generally, lack of resources such as time and funding were barriers,

Basically motivated, but at the moment I lack in time for proper research.

Dedicated time, time and time!

Funding is very limited for the research in my country.

Responses to how ESCP could support research involvement generated several themes, outlined in Table 4.

**Table 4:** Themes and illustrative quotes in relation to how ESCP could support research involvement

Theme	Illustrative quotes
Support through education and training	Offer relative, interactive and certified CPE workshops, seminars and/or events
	Develop online seminars about research topics/methods given by experts in the field
	Organisation of high-level research training classes (also at Ph.D. and post-doc level)
Support collaboration through networks and mentoring	A specific forum to help people who are interested to meet each other
	Establish a network and bringing together persons with a similar research interest(s)
	Opportunities for collaboration with colleagues having more experience or having experience in a complementary field
	Facilitate international research cooperation. Make a database of persons and related research activities and possibilities to work together in a (pan)-European context
Support through dissemination of research tools	Curate a collection of research tools for clinical pharmacy (e.g. questionnaires, scales, documentation guides

### ESCP symposium discussion group

Thirteen ESCP members participated in the focus group held during the symposium, representing Turkey (n = 3), France (n = 2), Qatar (n = 2), Switzerland (n = 2), Croatia (n = 1), Nigeria (n = 1), Sweden (n = 1), UK (n = 1).

Research findings are presented under two main headings of barriers and expectations.

#### Barriers to research aspirations ESCP members

Barriers to the clinical pharmacy research aspirations of the participants were categorised into five key themes and associated subthemes, as shown in Table 5.

**Table 5:** Barriers to research aspirations ESCP members

Themes	Subthemes
Insufficient collaboration	Limited opportunities to work across country borders
	Knowing who to collaborate with
	Finding collaborators based in other countries
	How to have different pharmacy sectors collaborating together
Lack of knowledge, skills and training	Need for training at all stages of research; development of ideas, data management, statistics, obtaining ethical approval, scientific writing and publication
	Lack of mentorship to enhance the development of advanced research skills
	Lack of expertise to ensure no key methodological flaws
	Difficulties in publishing if non-native English speaker
Unsupportive environment in practice	Unsupportive environment and lack of opportunities to participate in research
	Doing research on a hospital ward or community pharmacy where research is not a priority
	Lack of support to take research to the ‘next step’
	Difficulty in getting acceptance from international journals due to ‘local data’
	How to deal with breaks in the scientific career (e.g. due to family)
Insufficient time	Due to current workload
	Too much work
	How to maintain work life balance
Limited resources and funding opportunities	Not knowing where to access funding
	Identifying collaborators to develop funding applications with
	Lack of support for financial planning in writing grant applications

### Expectations from ESCP

There were five key themes and associated subthemes for their expectations from ESCP, as shown in Table 6.

**Table 6:** Expectations from ESCP

Themes	Subthemes
Mentorship	Early career expressed need for a mentor to provide support at all stages of the research process
	Could provide medium for mentoring support
Collaboration	Desire to collaborate internationally
	Promoted and supported by ESCP via encouraging international communication between researchers working in similar areas
	Support links between main research centres and possible exchange programmes Discussion forums to connect researchers/collaborators
	Promote research activities of ESCP members
	Could integrate research into the special interest groups
Education	Desire for related tutorials or guidelines for research activities
	Organization of intensive courses by ESCP to make researchers more competent methodologically
	Validated short education programs on research methodology for applying in different countries
Funding	To promote awareness of funding opportunities
	Collate funding opportunities, e.g. young researchers
	Support to encourage/develop applications for the funding calls
	Develop suite of templates e.g. proposal, consent, information leaflet
Publication	Practical sessions on writing for publication
	Support to write manuscripts that are more likely to be accepted in scientific journals

## Discussion

ESCP member respondents were highly involved in research, interested, experienced and confident. Responses to all three PCA components were positive, with the most negative for items on sufficient time and access to resources. Participants of the qualitative phase identified a number of areas in which ESCP could support their research aspirations through mentoring, supporting collaboration, and providing education and training.

Strengths to this research include the mixed methods approach and the use of a framework of behaviour change theories. The major limitation is the response rate, which was disappointing given that the study population comprised members of a professional network. There are therefore potential issues resulting from response bias with those responding potentially more interested and involved in research. It is notable that in excess of 40% of respondents were from an academic setting and 70% possessed a Ph.D. Further limitations include the potential lack of validity of self-reported data and social desirability bias. The quantitative results may therefore lack generalisability and the qualitative findings transferability to all ESCP members and the population of pharmacists in Europe.

It is, however, clear that there is a cohort of highly research active ESCP members involved in leading, supervising, publishing and presenting research to a much higher extent than in previous studies [7, 8]. This finding is not surprising given the high proportion of academics within the study respondents, for whom there would be expectations of research activities. There is the potential to harness activities of these individuals to augment research capacity, aligned to the aim of ESCP. As an international organisation, there is an opportunity for ESCP to support multicentre research to optimise the impact on patients, practitioners, organisations and society.

The summary scores for research interest were significantly higher than for experience confidence, as has been found in other studies of pharmacists [7, 8] and other health professionals [18–22]. However, the actual scores for ESCP respondents were much higher than in other studies. For example, a recent study of pharmacists in Scotland gave median summary scores of 50, 38.5 and 42 using the same scales for interest, experience and confidence respectively compared to 68, 62 and 64 in this study [8]. Again, these differences are likely to be attributed to the differences in characteristics of the participants on the studies.

The scores for the three PCA items were also higher than for the same items in the study in Scotland [8]. For the ‘support and opportunities to be involved in research’, respondents were very positive in terms of their research environment, and the support from the organisation and others. These responses may also reflect the academic setting of many respondents. The most negative responses were around time and resources for research, which are similar findings to many other studies [7, 8]. These issues were also reflected in the themes identified from the content analysis of open comments and the discussion groups. The responses for the ‘motivation for and outcomes of involvement in research’ were particularly positive, with respondents in agreement with the impact of research on patients, practitioners, organisations and themselves. For the ‘individual roles and characteristics around involvement in research’, there were positive responses around items of confidence, competence and enthusiasm. There are therefore many enablers to ESCP members being involved in research.

TDF domains identified as barriers to research can be used to aid the development of behaviour change interventions, defined as ‘coordinated sets of activities designed to change specified behaviour patterns’ [23, 24]. These interventions consist of interacting components known as ‘behaviour change techniques’ (BCTs), which are ‘observable and replicable components designed to change behaviour’ [23, 24]. While the lowest scoring items of time and resources map to the TDF domain of ‘environmental context and resources’, the BCTs relate more to aspects of culture and require intervention at the organisational, leadership and management levels. Even though ESCP is not the employing organisation for the members, there is opportunity to have an influence, as identified in the qualitative element of the study.

Future research can centre around ESCP developing mechanisms to support these aspects followed by evaluation of quantitative (e.g. research activity and output) and qualitative (e.g. members’ perspectives) outcomes. In addition, there may be merit in focusing on those from non-academic settings.

## Conclusion

ESCP participants in this study were highly research active, interested, experienced, confident and positive regarding research conduct and outcomes. There is an opportunity for ESCP to harness these activities, aligned to the ESCP aim, and provide support in the form of mentoring, education and training, and facilitating research collaboration.
